# Few-Shot Learning for Clinical Natural Language Processing Using Siamese Neural Networks: Algorithm Development and Validation Study

**DOI:** 10.2196/44293

**Published:** 2023-05-04

**Authors:** David Oniani, Premkumar Chandrasekar, Sonish Sivarajkumar, Yanshan Wang

**Affiliations:** 1 Department of Health Information Management University of Pittsburgh Pittsburgh, PA United States; 2 Intelligent Systems Program University of Pittsburgh Pittsburgh, PA United States; 3 Department of Biomedical Informatics University of Pittsburgh Pittsburgh, PA United States; 4 Clinical and Translational Science Institute University of Pittsburgh Pittsburgh, PA United States

**Keywords:** few-shot learning, FSL, Siamese neural network, SNN, natural language processing, NLP, neural networks

## Abstract

**Background:**

Natural language processing (NLP) has become an emerging technology in health care that leverages a large amount of free-text data in electronic health records to improve patient care, support clinical decisions, and facilitate clinical and translational science research. Recently, deep learning has achieved state-of-the-art performance in many clinical NLP tasks. However, training deep learning models often requires large, annotated data sets, which are normally not publicly available and can be time-consuming to build in clinical domains. Working with smaller annotated data sets is typical in clinical NLP; therefore, ensuring that deep learning models perform well is crucial for real-world clinical NLP applications. A widely adopted approach is fine-tuning existing pretrained language models, but these attempts fall short when the training data set contains only a few annotated samples. Few-shot learning (FSL) has recently been investigated to tackle this problem. Siamese neural network (SNN) has been widely used as an FSL approach in computer vision but has not been studied well in NLP. Furthermore, the literature on its applications in clinical domains is scarce.

**Objective:**

The aim of our study is to propose and evaluate SNN-based approaches for few-shot clinical NLP tasks.

**Methods:**

We propose 2 SNN-based FSL approaches, including pretrained SNN and SNN with second-order embeddings. We evaluate the proposed approaches on the clinical sentence classification task. We experiment with 3 few-shot settings, including 4-shot, 8-shot, and 16-shot learning. The clinical NLP task is benchmarked using the following 4 pretrained language models: bidirectional encoder representations from transformers (BERT), BERT for biomedical text mining (BioBERT), BioBERT trained on clinical notes (BioClinicalBERT), and generative pretrained transformer 2 (GPT-2). We also present a performance comparison between SNN-based approaches and the prompt-based GPT-2 approach.

**Results:**

In 4-shot sentence classification tasks, GPT-2 had the highest precision (0.63), but its recall (0.38) and *F* score (0.42) were lower than those of BioBERT-based pretrained SNN (0.45 and 0.46, respectively). In both 8-shot and 16-shot settings, SNN-based approaches outperformed GPT-2 in all 3 metrics of precision, recall, and *F* score.

**Conclusions:**

The experimental results verified the effectiveness of the proposed SNN approaches for few-shot clinical NLP tasks.

## Introduction

### Background

Deep neural networks (DNNs), due to their performance [1], currently dominate both computer vision and natural language processing (NLP) literature. However, fully using the capabilities of DNNs requires large training data sets. To tackle this problem, researchers have tried to reduce the complexity of the DNN models to obtain comparable performance when the training data set is small [2]. The few-shot learning (FSL) paradigm is an alternative attempt that aims to improve model performance under data constraints. The goal of FSL is to efficiently learn from a small number of *shots* (ie, data samples or instances). The number of samples usually ranges from 1 to 100 per class [3,4]. There is a growing interest in the artificial intelligence (AI) research community in FSL, and several different strategies have been developed for FSL, including Bowtie Networks [5], Induction Networks [6], and Prototypical Networks [7].

A Siamese neural network (SNN), sometimes called a twin neural network, is an artificial neural network that uses 2 parallel, weight-sharing machine learning models to compute comparable embeddings. The SNN architecture has shown promising results as an FSL approach in computer vision for similarity detection [8] and duplicate identification [9]. Yet, its usage in NLP has been understudied, and, to the best of our knowledge, there have not been any studies investigating SNNs for clinical NLP.

In SNNs, neural networks are trained to compute embeddings. In NLP, deep learning has achieved state-of-the-art performance since it could generate comprehensive embeddings to encode semantic and syntactic information. The primary use of deep learning in NLP is to represent the language in a vectorized form (ie, embeddings) so that the representation can be used for different NLP tasks, such as natural language generation, text classification, and semantic textual similarity. Thus, having a robust embedding-generation mechanism is crucial for most NLP tasks. Since the context of words, sentences, and more generally, text is important to learn meaningful embeddings, context-aware embedding-generation models, such as bidirectional encoder representations from transformers (BERT) [10], often show promising results. Furthermore, depending on the domain, the context also varies. For this purpose, researchers and engineers have built domain-specific, specialized models for use in downstream tasks. Examples of such models include BERT for biomedical text mining (BioBERT) [11] trained from biomedical literature texts and Bio + clinical BERT (BioClinicalBERT) trained from clinical texts [12]. However, leveraging contextual embeddings for FSL has rarely been studied in clinical NLP.

FSL is critical for clinical NLP as annotating a large training data set is costly and usually requires involving domain experts. On the other hand, it is common to have a few clinical text samples annotated by physicians. One example could be clinical notes with annotations of a rare disease, with the number of samples limited due to the nature of the disease. Despite such challenges, the importance of using AI in clinical applications cannot be understated. AI could assist physicians in their decision-making, facilitate clinical and translational research, and significantly reduce the need for manual work. This study proposes an FSL approach based on SNNs to tackle clinical NLP tasks with only a few annotated training samples. Two SNN-based FSL approaches are proposed: pretrained SNN (PT-SNN) and SNN with second-order embeddings (SOE-SNN). Both approaches used the 3 different transformer models of BERT, BioBERT, and BioClinicalBERT. We evaluated the proposed strategies on the clinical sentence classification task. Clinical text classification refers to the classification of clinical sentences based on predefined classes. We show that SNN-based methods outperform the baseline, generative pretrained transformer 2 (GPT-2) model in few-shot settings for the task. Finally, we discuss the limitations and future work.

### Related Work

There have been studies evaluating the usability of SNNs for image classification. Li et al [13] used SNNs for the classification of high-dimensional radiomic features extracted from MRI images. Hunt et al [14] applied SNNs for the classification of electrograms. Zhao et al [15] have used SNNs for hyperspectral image classification.

In sentence classification, Reimers and Gurevych [16] used SNNs to derive semantically meaningful sentence embeddings that can be compared using cosine similarity. It is important to note that the package we used in our experiments to generate embeddings was based on this paper [16]. However, the primary goal of our experiments was not generating sentence embeddings, but rather designing techniques for using such embeddings in few-shot clinical sentence classification tasks.

In the context of FSL, SNNs have been used by Torres et al [17] for one-shot, convolutional neural networks–based classification to optimize the discovery of novel compounds based on a reduced set of candidate drugs. Droghini et al [18] employed SNNs for few-shot human fall detection purposes using images. However, none of these studies used SNN-based FSL for NLP.

In few-shot text classification, Wei et al [19] used data augmentation to improve the performance of triplet networks. Liu et al [20] proposed distribution estimation to augment the labeled samples by sampling from the estimated distribution. Wang et al [21] represented each task using gradient information from a base model and trained an adaptation network that modulates a text classifier conditioned on the task representation.

There is only a recent study by Müller et al [22] that explored SNNs for FSL in NLP and demonstrated the high performance of pretrained SNNs that embed texts and labels. To the best of our knowledge, none of the studies referenced above are using SNNs to perform FSL in the clinical NLP domain.

## Methods

### Ethical Considerations

As the study is using a publicly available data set that is accessible under the data use agreement, there is no requirement for an institutional review board.

### Data Set Derived from the Medical Information Mart for Intensive Care

The sentences were obtained from the Medical Information Mart for Intensive Care (MIMIC-III) database [23]. We used the same data set as in the HealthPrompt paper by Sivarajkumar and Wang [24], but with classes suitable for 4-shot, 8-shot, and 16-shot FSL experiments. In total, the data set had 444 samples and 4 classes. Table 1 shows the distribution of classes in the data set.

Since we had 444 samples in total and performed 4-, 8-, and 16-shot experiments, the train size varied and was 16, 32, and 64 samples with the test sizes of 428, 412, and 380 samples, respectively.

**Table 1:** Few-shot sentence classification data set (N=444).

Label	Sample, n (%)
ADVANCED.LUNG.DISEASE	245 (55.2)
ADVANCED.HEART.DISEASE	117 (26.4)
CHRONIC.PAIN.FIBROMYALGIA	48 (10.8)
ADVANCED.CANCER	34 (7.7)

### Sentence-Level Embeddings

For generating contextual, sentence-level embeddings, we used the sentence-transformers package [25]. The package provides intuitive and easy-to-use methods for computing dense vector representations of sentences, paragraphs, and images. The models are based on transformers such as BERT, RoBERTa [26], and so on, and achieve state-of-the-art performance in various tasks. The generated embeddings are such that similar texts are close in the latent space and can efficiently be found using cosine similarity. Thus, for sentences *a* and *b* with the corresponding embeddings *A* and *B*, we can compute the cosine similarity as follows:

cosine similarity(A, B) = (A ⋅ B) / (||A||_2_ ||B||_2_) **(1)**

### Model Architecture

**Figure 1:**
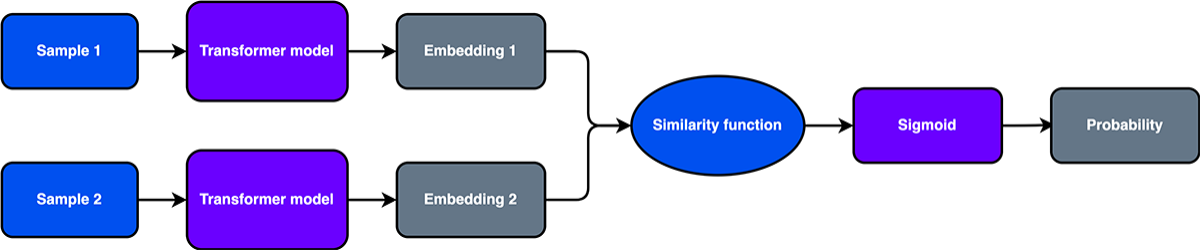
Siamese neural network (SNN) architecture.

The SNN’s architecture leverages 2 parallel weight-sharing machine learning models (Figure 1). In the forward pass, 2 samples are passed into the models and mapped down to the latent space. The embeddings in the latent space are then compared using a similarity function, as shown in Equation 2. The similarity function is a hyperparameter that can vary based on the task and could range from Euclidean distance to Manhattan distance or cosine similarity. Depending on the similarity function, the similarity value can then be mapped onto the (0*,* 1) interval by applying the Sigmoid function. Finally, a high similarity value means that the input samples likely belong to the same category and vice versa.

out = σ(distance(*emb*_1_, *emb*_2_)) **(2)**

During training, SNN conducts representation learning [27] and attempts to have the best approximation for the input embeddings. The representation is learned by penalizing the loss if the model yields a high similarity value for inputs from different classes or if the model yields a low similarity value for inputs from the same class.

The SNN architecture naturally allows for data augmentation. For instance, in the case of 8-shot learning, the traditional training approach would involve passing 8 samples directly into the model. This approach is very limiting with such a small number of samples. SNN takes a different route and instead considers unique comparisons within the training set. With the training set consisting of 8 samples, there are 8 ∗ 7 / 2 = 28 unique comparisons. Thus, instead of 8 training samples, we get 28, which is 3.5 times more. In the case of 16 samples, the improvement is even more significant as the number of unique comparisons is 120, and there is a 7.5-fold data augmentation.

More generally, under *N*-way-*K*-shot classification settings, for the data set *D*_train_ with *N* class labels and *K* labeled samples for each class, the following holds after SNN-style augmentation:

D_train SNN_ = {(x_i_, x_j_) | x_i_, x_j_ ∈ D_train_, i < j} **(3)**

size(D_train SNN_) = ((NK)^2^ – NK) / 2 **(4)**

### Pretrained SNN

In the first approach, we leverage the pretrained language models (PLMs) to generate embeddings for the SNN, called pretrained SNN (PT-SNN). We used 3 PLMs in this approach, namely BERT, BioBERT, and BioClinicalBERT, to generate embeddings for the input training samples.

In the following, we illustrate how to use the PT-SNN for classification. Suppose we want to perform binary classification. We are given 2 classes *C*_1_ and *C*_2_, a training set *D*_train_, and a testing set *D*_test_. We first compute embeddings for all samples in both *D*_train_ and *D*_test_. For every testing sample, using the generated embeddings, we compute the similarity with respect to every training sample and compute the mean similarity values for classes. For instance, mean similarity value for some samples *x ∈ D*_test_ with respect to *C*_1_ and *C*_2_ might be 0.2 and 0.6, respectively. Since 0.6 is greater than 0.2, we classify sample *x* as being in class *C*_2_. It should be noted that the algorithm is similar to the k-nearest neighbors [28] classification algorithm.

Note that our classification approach is such that using Sigmoid is not necessary. In the case of SOE-SNN, it is required during training, but not during testing (See Algorithm 1 in Textbox 1).

Algorithm 1 presents the pseudocode for the classification algorithm and the evaluation approach. Here, *EvalIters* refers to the number of averaging iterations for addressing the instability issues. In our case, *EvalIters* is 3.

**Algorithm 1—our proposed algorithm for Siamese neural network–style classification and evaluation for few-shot learning.**

Require: *D_train_*: Train data set

Require: *E_test_*: Test data set embeddings

Require: *L_test_*: Test data set labels

Require: *EvalIters*: Number of evaluation iterations

Require: *RandSubset*: A function that randomly subsets a data set with the given seed

Require: *L*1*Normalize*: L1-normalizes the input tensor

Require: *L*2*Normalize*: L2-normalizes the input tensor

Require: *Arange*: Constructs a tensor of numbers from the given start and end (exclusive) with the step size of one

Require: *Argmax*: Finds the index of the maximum value along the given dimension

Require: *ComputeMetrics*: Computes evaluation metrics: precision, recall, and *F* score

Require: *MatMul*: Performs a matrix multiplication of the given tensors

Require: *Max*: Finds the maximum value of all elements in the input tensor

Require: *Mean*: Calculates the mean of a vector along the given dimension

Require: *NumElements*: Finds the number of elements in the input tensor

Require: *Transpose*: Transposes the input tensor

Require: *Zeros*: Creates the tensor of zeros with the given dimensions

     1: *Metrics ← Zeros*(*EvalIters,* 3);

     2: for *Idx ←* 0 to *EvalIters* do

     3: *E_train_, L_train_ ← RandSubset*(*D_train_, Seed* = *Idx*);

     4: L2*_train_ ← L*2*Normalize*(*E_test_*);

     5: L2*_test_ ← L*2*Normalize*(*E_train_*);

     6: *SimilarityTable ← MatMul*(*L*2*_test_, Transpose*(*L*2*_train_*));

     7: *LabelTable ← Zeros*(*Max*(*L_train_*) + 1*, NumElements*(*L_train_*));

     8: *LabelTable* [*L_train_, Arange*(*NumElements*(*L_train_*))] *←* 1;

     9: *LabelTable ← L*1*Normalize*(*LabelTable*);

     10: *Out← Argmax*(*MatMul*(*SimilarityTable, Transpose*(*LabelTable*))*, Dim* = 1);

     11: *Metrics*[*Idx*] *← ComputeMetrics*(*L_test_, Out*);

     12: end for

     13: *Precision, Recall, Fscore ← Mean*(*Metrics, Dim* = 0).

We use the vectorized implementations of cosine similarity, group by, and aggregate operations described in Multimedia Appendix 1.

Such a strategy for classification can be slow in cases where the training set is large. However, the proposed approach is feasible in the FSL settings, where the number of annotated samples is limited. Thus, we do not expect significant performance drawbacks when the number of samples is not large. Furthermore, the proposed PT-SNN approach can be high-performing under FSL settings.

We have also released a codebase implementing the proposed algorithms and models [29].

### SNN With Second-Order Embeddings

**Figure 2:**
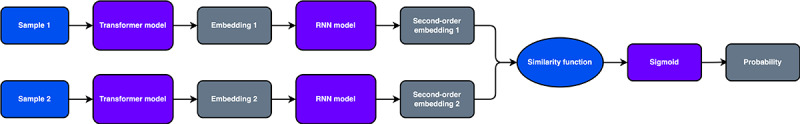
Siamese neural network with second-order embeddings (SOE-SNN) architecture. RNN: recurrent neural network.

The second proposed approach is SOE-SNN where we apply an additional recurrent neural network (RNN) layer, such as long-short term memory or gated recurrent unit to the generated embeddings and then train the SNN model in the fashion described in the model architecture section (Figure 2). In our experiments, we used bidirectional long-short term memory for producing second-order embeddings.

Specifically, we first obtain the embeddings for all training samples from the PLMs. Half of the samples are used for training the RNN, and the other half is used for the classification algorithm described in Algorithm 1. For the RNN half, all possible unique pairs of the samples are generated and labeled 1 if the samples in the pair are of the same class or 0 if they come from different classes. Binary cross entropy [30] and AdamW [31] are used as the loss function and the optimizer, respectively. The loss function and the optimizer were used for training the RNN. Similar to the PT-SNN, the transformer model that generates embeddings was not updated, and as such, one could think of this as a frozen component of the training pipeline.

Model evaluation is done in the same manner as in PT-SNNs, where we compute mean similarity scores and average out the metrics over 3 evaluation iterations to handle the potential instability issues.

### FSL Model Evaluation

Systematically evaluating FSL model performance can be tricky since fine-tuning or making predictions on small data sets could potentially suffer from instability [32]. To address this issue, we propose the averaging strategy for model evaluation. For every few-shot experiment (eg, 4-shot, 8-shot, and 16-shot experiments), we use randomized sampling to sample 4, 8, or 16 samples per class and create a training data set. We perform this *M* times, and therefore, for every experiment, *M* randomly generated training sets are evaluated on the test set. Finally, the metrics are averaged out and reported as the final scores.

Metric = (Σ_i=1_^M^ Metric_i_) / M **(5)**

Such an approach gives a more robust view of the model’s performance in possibly unstable scenarios. Therefore, we choose *M*=3 and employ this strategy in all reported metrics. As for metrics, we choose precision, recall, and *F* score.

### Baseline Model

Despite the availability of newer GPT models such as ChatGPT and GPT-4, they cannot be used on the MIMIC data set as per the terms of the data use agreement. Therefore, we used the open source GPT-2. We used the GPT-2 [33] with 355 million parameters as the baseline model. We obtained 4, 8, and 16 samples per class to generate predictions. To achieve this, we used the transformers package [34]. Note that no fine-tuning was done in this case, and instead, the existing GPT-2 model was used directly for generating responses.

We used a prefix prompt with all possible classes appended to the sentence for classification, followed by the incomplete sentence that would have to be completed by GPT-2. The proposed prompt is similar to the cloze prompt that showed the best performance in Sivarajkumar and Wang [24]. We modified the prompt by adding additional information at the end of the text (all 4 labels) and moved the mask at the end, effectively turning it into a prefix prompt. Thus, we used the following prompt:

{text}. options are advanced cancer, advanced heart disease, advanced lung disease, chronic pain fibromyalgia. type of disease {mask}

where {text} is the input text and {mask} is the placeholder for GPT-2 to fill in with the generated text. Appending the list of labels to the end of the input text was done to help the GPT-2 model by showing all available options. We used the maximum context size of 1024—the most GPT-2 can handle. If the total number of tokens exceeded 1024, the sentence was trimmed from the end to keep the prompt intact.

Finally, the generated responses were analyzed and evaluated by the annotator. The annotator labeled every GPT-2 response with the semantically closest class (1 of 4 options). Note that the annotator evaluated the responses only once. Thus, for GPT-2, the number of averaging iterations is 1 (ie, *M*=1).

## Results

We present the results of 4-shot, 8-shot, and 16-shot experiments for few-shot sentence classification task. We used models based on BERT, BioBERT, BioClinicalBERT. The results are shown in Table 2.

In the 4-shot sentence classification task, the baseline, GPT-2 model had the highest precision (0.63). BioClinicalBERT-based SOE-SNN came next with a precision score of 0.57. PT-SNN had the highest recall and *F* score values of 0.45 and 0.46, respectively. BioClinicalBERT-based PT-SNN was the second with recall and *F* score of 0.42 and 0.43, respectively. Thus, in 4-shot settings, GPT-2 had a higher precision, but its recall and *F* score were lower than those of SNN-based approaches.

In 8-shot experiments, BioClinicalBERT-based PT-SNN outperformed all other approaches in precision, with a value of 0.64. BioBERT-based SOE-SNN had both the highest recall and the highest *F* score of 0.50 and 0.53, respectively. GPT-2 did not have the highest score in any of the metrics. Hence, for 8-shot learning, SNN-based approaches outperformed GPT-2.

As for 16-shot learning, BioClinicalBERT-based SOE-SNN had the highest precision value of 0.70. BioBERT-based PT-SNN had the highest recall (0.55), and BioClinicalBERT-based PT-SNN had the highest *F* score (0.58). GPT-2 did not have the highest score in any of the metrics, with most models having higher precision, recall, and *F* score. Overall, SNN-based approaches outperformed the baseline GPT-2 model.

**Table 2:** Few-shot sentence classification.

Approach	Model	Shots	Precision	Recall	*F* score
GPT-2^a^	GPT-2	4	*0.63*	0.38	0.42
PT-SNN^b^	BERT^c^	4	0.49	0.37	0.37
PT-SNN	BioBERT^d^	4	0.53	*0.45*	*0.46*
PT-SNN	BioClinicalBERT^e^	4	0.50	0.42	0.43
SOE-SNN^f^	BERT	4	0.49	0.26	0.30
SOE-SNN	BioBERT	4	0.52	0.19	0.17
SOE-SNN	BioClinicalBERT	4	0.57	0.27	0.24
GPT-2	GPT-2	8	0.63	0.38	0.42
PT-SNN	BERT	8	0.62	0.45	0.47
PT-SNN	BioBERT	8	0.61	0.48	0.50
PT-SNN	BioClinicalBERT	8	*0.64*	0.44	0.49
SOE-SNN	BERT	8	0.55	0.43	0.46
SOE-SNN	BioBERT	8	0.61	*0.50*	*0.53*
SOE-SNN	BioClinicalBERT	8	0.58	0.32	0.32
GPT-2	GPT-2	16	0.65	0.38	0.42
PT-SNN	BERT	16	0.64	0.51	0.52
PT-SNN	BioBERT	16	0.65	*0.55*	0.56
PT-SNN	BioClinicalBERT	16	0.69	0.54	*0.58*
SOE-SNN	BERT	16	0.59	0.44	0.48
SOE-SNN	BioBERT	16	0.43	0.38	0.36
SOE-SNN	BioClinicalBERT	16	*0.70*	0.39	0.38

^a^GPT-2: generative pretrained transformer 2.

^b^PT-SNN: pretrained Siamese neural network.

^c^BERT: bidirectional encoder representations from transformers.

^d^BioBERT: bidirectional encoder representations from transformers for biomedical text mining.

^e^BioClinicalBERT: Bio + clinical bidirectional encoder representations from transformers.

^f^SOE-NN: Siamese neural network with second-order embeddings.

## Discussion

### Limitations and Future Work

There are several limitations of the work that can be addressed by further exploring FSL and SNNs. First, we did not compare the results to traditional baseline models such as support vector machine, logistic regression, multinomial logistic regression, random forest, and so on. Second, other data sets could also be used for evaluating the performance of SNNs in text classification. Third, since we can perform sentence-level classification, another interesting research direction could be document classification, where a document can be modeled as a collection of sentences. Fourth, in the SOE-SNN, since we only experiment with 1 splitting strategy (half for fine-tuning embeddings and half for classification and evaluation), other RNN training versus classification ratios can also be noteworthy. Additionally, it is important to note that data sets for FSL, especially clinical FSL, are difficult to find. Ge et al [35] have emphasized that “(68%) studies reconstructed existing datasets to create few-shot scenarios synthetically.” Thus, building a brand-new FSL data set and then evaluating the performance of the proposed methods could also be an interesting future research direction.

### Conclusion

We conducted few-shot learning experiments evaluating the performance of SNN models on the clinical sentence classification task. The SNN models were based on transformer models—BERT, BioBERT, and BioClinicalBERT. Since performance evaluation on small data sets may suffer from instability, a special evaluation strategy was used. We conclude that, overall, SNN-based models outperformed the baseline GPT-2 model for sentence classification tasks. The limitations of the work have also been discussed alongside potential future directions of research.

## Appendix

Multimedia Appendix 1 Vectorized cosine similarity, group by, and aggregate.

## Abbreviations

AI: artificial intelligence
BERT: bidirectional encoder representations from transformers
BioBERT: bidirectional encoder representations from transformers for biomedical text mining
BioClinicalBERT: Bio + clinical bidirectional encoder representations from transformers
DNN: deep neural networks
FSL: few-shot learning
GPT-2: generative pretrained transformer 2
MIMIC-III: Medical Information Mart for Intensive Care
NLP: natural language processing
PLM: pretrained language model
PT-SNN: pretrained Siamese neural network
RNN: recurrent neural network
SNN: Siamese neural network
SOE-SNN: Siamese neural network with second-order embeddings
