# Supplementary material for: Few-Shot Learning for Clinical Natural Language Processing Using Siamese Neural Networks: Algorithm Development and Validation Study
Source: JMIR AI. 2023 May 4;2:e44293. doi: 10.2196/44293 (PMC11041484; doi:10.2196/44293)
Supplement: Multimedia Appendix 1 [file ai_v2i1e44293_app1.doc]

## ***Multimedia Appendix 1. Vectorized Cosine Similarity, Group By, and Aggregate***

The performance of the algorithm can have a direct impact on training and serving time. Hence, it is important to ensure that the inference pipeline is efficient. Many of the AI/ML frameworks, such as PyTorch (that we used in our work), are implemented in low-level programming languages such as C and C++. Additionally, these frameworks provide a variety of highly optimized tensor operations that use vectorization and Single Instruction, Multiple Data (SIMD) processing. Properly utilizing these capabilities often requires thinking about ways to transform the imperative-style code into the declarative-style one, which in turn requires abstracting away for loops, if statements, and other control flow mechanisms through optimized tensor operations.

This section discusses implementations for vectorized cosine similarity, group by, and aggregate operations. These three operations will help compute the mean cosine similarity per label, which is at the core of the proposed classification algorithm and can be considered a hot path of the program. Note that some operations use broadcasting (available in libraries such as PyTorch and NumPy).

## ***Cosine Similarity***

Suppose we have a collection of two text embeddings *U* and *V*. Our task is to compute cosine similarity values of embeddings in the collection *U* with respect to those in the collection *V*. Now, expanding the cosine similarity formula, notice that the following holds:

cosine similarity(*U*, *V*) = (*U* ⋅ *V*) / (||*U*||2 ||*V*||_2_) = (*U* / ||*U*||_2_) ⋅ (*V* / ||*V*||_2_) **(1)**

While the first formulation of cosine similarity defines it as the dot product over the product of L2 norms, the latter represents it as the dot product of vectors over L2 norms. Using the dot product of fractions representation, cosine similarity can be intuitively implemented as follows:

**Algorithm 1** Vectorized Cosine Similarity Implementation

**Require:** *U*: A collection of embeddings

**Require:** *V*: A collection of embeddings

**Require:** *L*2*Normalize*: L2-normalizes the input tensor

**Require:** *MatMul*: Performs matrix multiplication of the given tensors

**Require:** *Transpose*: Transposes the input tensor

1: *L*2*_U_ ← L*2*Normalize*(*U*);

2: *L*2*_V_ ← L*2*Normalize*(*V*);

3: *SimilarityTable ← MatMul*(*L*2*_U_, Transpose*(*L*2*_V_*));

## ***Group By***

Grouping by a label is an operation that is also necessary for implementing the proposed algorithms efficiently.

**Algorithm 2** Vectorized Group By Implementation

**Require:** *U*: A collection of labels with respect to which we want to do grouping

**Require:** *L*1*Normalize*: L1-normalizes the input tensor

**Require:** *Arange*: Constructs a tensor of numbers from the given start and end (exclusive) with the step size of one
**Require:** *Max*: Finds the maximum value of all elements in the input tensor
**Require:** *NumElements*: Finds the number of elements in the input tensor
**Require:** *Zeros*: Creates the tensor of zeros with the given dimensions

1: *LabelTable ←* *Zeros*(*Max*(*U*) + 1*, NumElements*(*U*));

2: *LabelTable*[*U, Arange*(*NumElements*(*U*))] *←* 1;

3: *LabelTable ← L*1*Normalize*(*LabelTable*);

In Algorithm 2 above, we first initialize the tensor of zeros, where the number of rows equals the number of labels (1 is added to the maximum value since we start labeling with 0) and the number of columns equals the number of elements in the collection for grouping. This procedure builds a table to be populated with weights. Using broadcasting, we then obtain all rows by indexing into the label table by the collection and setting 0s to 1s. We finally perform L1 normalization of the table to have the group-by weights.

## ***Aggregate***

After having both cosine similarity values and the group-by table, we can compute the similarity values based on a label using matrix multiplication.

**Algorithm 3** Vectorized Aggregate Implementation

**Require:** *SimilarityTable*: A cosine similarity table

**Require:** *LabelTable*: A table holding weights for grouping by label

**Require:** *Argmax*: Finds the index of the maximum value along the given dimension

**Require:** *MatMul*: Performs matrix multiplication of the given tensors

**Require:** *Transpose*: Transposes the input tensor

1: *Out ←* *Argmax*(*MatMul*(*SimilarityTable,* *Transpose*(*LabelTable*))*, Dim* = 1);

Algorithm 3 above computes a tensor that holds the most similar label for every given input sample.
